# Implicit action encoding influences personal-trait judgments^☆^

**DOI:** 10.1016/j.cognition.2005.11.003

**Published:** 2007-02

**Authors:** Patric Bach, Steven P. Tipper

**Affiliations:** Centre for Clinical and Cognitive Neuroscience, University of Wales, Bangor, Gwynedd LL57 2AS, UK

**Keywords:** Vision–action compatibility, Mirror neurons, Personal-trait judgments, Autism

## Abstract

When an observed action (e.g., kicking) is compatible to a to be produced action (e.g., a foot-key response as compared to a finger-key response), then the self-produced action is more fluent, that is, it is more accurate and faster. A series of experiments explore the notion that vision–action compatibility effects can influence personal-trait judgments. It is demonstrated that when an observed individual carries out an action that is compatible with the participants’ response, (1) this individual is identified more fluently, and (2) the observed individual’s personality is attributed with the properties of the observed action. For example, if it is easier to identify one individual with a foot-response when he is seen kicking a ball, as compared to typing, he is perceived to be more ‘sporty’. In contrast, if it is easier to identify one individual with a finger response when he is seen typing as compared to kicking a ball, he is associated with the ‘academic’ trait. These personal-trait judgment effects can be observed with explicit measures, where participants are asked to rate the sporty/academic nature of the person on a scale. They are also obtained when implicit measures are taken in a priming task, where participants are never explicitly asked to rate the personalities of the individuals. A control experiment rules out that these personal-trait effects are merely due to an association of motor responses (foot, finger) to individuals while identifying them, but that these effects depend on a prior manipulation of vision–action fluency.

## 1 Introduction

There is mounting evidence that human cognition – social and other – does not rely on amodal representations, but is ‘grounded’ in the perceptual and motor systems. Accordingly, the representations of an entity or an event consist of the relevant perceptual and motor states that were present when these things were experienced (e.g., Barsalou, 1999, 2003; Glenberg, 1997; Zwaan, Stanfield, & Yaxley, 2002).

Perceived actions, in particular, seem to activate the representations an observer would use to produce the same actions (e.g., Hommel, Müsseler, Aschersleben, & Prinz, 2001; Iacoboni, 2005). It has been found that actions are more fluent – faster and more accurate – when the actor concurrently perceives another person carry out the same action (e.g., Brass, Bekkering, Wohlschläger, & Prinz, 2000; Castiello, 2003; Fadiga, Fogassi, Pavesi, & Rizzolatti, 1996; Kilner, Paulignan, & Blakemore, 2003; Stürmer, Aschersleben, & Prinz, 2000). Neurophysiological findings provided a neuronal substrate for these processes. DiPellegrino and colleagues have discovered neurons in the macaque premotor cortex that fire if the monkey produces a particular action, but also if it observes another individual produce the same action (DiPellegrino, Fadiga, Fogassi, Gallese, & Rizzolatti, 1992). Evidence for this so-called ‘mirror system’ is now well established in monkey (Gallese, Fadiga, Fogassi, & Rizzolatti, 1996) and human (e.g., Buccino et al., 2001; Grèzes, Armony, Rowe, & Passingham, 2003; Rizzolatti, Fadiga, Gallese, & Fogassi, 1996).

The notion that perceived actions were ‘matched directly’ (Rizzolatti, Fogassi, & Gallese, 2000) to the corresponding action representations of the observer had a significant impact on research in social cognition. Perception–action matching processes may form the basis of observational learning (e.g., Elsner & Aschersleben, 2003; Liberman & Mattingly, 1985; Stefan et al., 2005) and are also critical for smooth and coherent social intercourse. Humans mimic the gestures, body postures, and facial expression of the persons they interact with. Although completely unconscious and non-strategic, this mimicking behavior facilitates social interactions and bonding between people (e.g., Chartrand & Bargh, 1999). For instance, when Van Baaren, Holland, Steenaert, and van Knippenberg (2003) required a waitress to either mimic (repeat back) or not mimic a food order made by a customer, the level of tips received was significantly higher in the mimicking case (see Van Baaren, Holland, Kawakami, & van Knippenberg, 2004 for other examples of pro-social behavior evoked by action mimicking).

According to embodied and motor accounts of social cognition (e.g., Barsalou, Niedenthal, Barbey, & Ruppert, 2003; Blakemore & Decety, 2001; Preston & de Waal, 2002; for a critique, see Jacob & Jeannerod, 2005) these findings imply that observers ‘simulate’ the bodily states of other persons on the basis of their own sensorimotor systems. By putting themselves into the shoes of others, they gain empathic insights into these persons’ personalities, goals, and emotional states (e.g., Gallese, 2001, 2003; Preston & de Waal, 2002; for a neuronal mechanism that could drive such inferences, see Barsalou et al., 2003). Perception–action matching mechanisms might therefore also form the basis of higher social cognitive functions, such as intention reading, Theory Of Mind, or the attribution of emotional states and personal traits to other persons (e.g., Blakemore & Decety, 2001; Gallese & Metzinger, 2003; Iacoboni, 2005). A malfunctioning mirror system might also underlie the social deficits of the autistic disorder (e.g., Nishitani, Avikainen, & Hari, 2004; Williams, Whiten, Suddendorf, & Perrett, 2001).

Consistent with such simulation accounts of social perception, it has been found that empathic people exhibit more mimicking behavior than non-empathic people (e.g., Sonnby-Borgström, Jönsson, & Svensson, 2003). Similarly, lesion and imaging studies show that partially overlapping brain areas process facial expressions and painful experiences of self and other (e.g., Adolphs, Damasio, Tranel, Cooper, & Damasio, 2000; Morrison, Lloyd, DiPellegrino, & Roberts, 2004), and there is evidence that an intact premotor cortex (i.e., mirror system) is required to attribute personality traits via observation of a person’s actions (Heberlein, Adolphs, Tranel, & Damasio, 2004). The notion that the mirror system was impaired in autism has also been supported by recent studies. In contrast to healthy subjects, autistic individuals do not always exhibit motor facilitation during action observation (Théoret et al., 2005).

The aim of the present work was to provide direct evidence for vision–action matching accounts of social perception by demonstrating that the attribution of personal traits relies on processes in the observer’s action system. Our experimental paradigm rests on the following notion: if the observer’s action system is involved in social perception, then manipulations that affect the action system of the observer should also influence how other persons are viewed. Similar research strategies have been applied before to show that the state of the observer’s action system affects, for instance, the perceived movement direction of illusory revolving figures (Wohlschläger, 2000), the perceived weight of a box lifted by another person (Hamilton, Wolpert, & Frith, 2004), or the perceived movement speed of point light walkers (Jacobs & Shiffrar, 2005). Similar techniques have also been applied in research on embodied (social) cognition. For instance, Strack, Martin, and Stepper (1988) have shown that manipulations that induce smiles or frowns also affect how funny the participants rated cartoons they perceived at the same time (for a review of related findings, see Barsalou et al., 2003). Here, we extend this logic to investigate whether the fluency states of the observer’s action system becomes associated with the actions other people carry out at the same time, and hence influence how the personality of these people is perceived.

Consider the following experimental situation. The participant’s task is to identify two individuals: if it is George, press a key with the right index finger, if John, press a foot key with the right foot. The individuals are presented carrying out either a sporty action (kicking a ball) or an academic action (typing on a keyboard). It is predicted that the depicted actions should influence the fluency with which the foot- and finger-key responses are executed, even though they are irrelevant to the task of person identification. Thus, the right finger-key press response to identify George will be faster and more accurate when he is carrying out the academic action (typing) than when he is carrying out the sporty action (kicking a ball); the opposite pattern will be observed for the right foot-key response to identify John.

Such vision–action compatibility effects would confirm prior studies showing that observed actions and to be executed responses rely on overlapping representations in the observer’s action system (cf. Brass et al., 2000; Stürmer et al., 2000). What is novel about our approach is the idea that the manipulation of vision–action compatibility might also influence what kind of people ‘George’ and ‘John’ are perceived to be. That is, because the finger-key press to identify ‘George’ is more fluent when he is carrying out the academic action than when carrying out the sporty action, it is predicted that participants will report that he is a more academic than sporty person. In contrast, because the foot response to identify ‘John’ will be more fluent when he is carrying out the sporty action of kicking a ball than when carrying out the academic action of typing, the participants will rate him as more sporty than academic.

To briefly preview our findings: it is indeed the case that the fluency of the participants’ responses affected the attribution of personal traits to the individuals. These personality-trait judgment effects can be observed with both explicit measures where participants are asked to rate the sporty/academic nature of George and John on a scale, and when implicit measures are taken in a priming task where participants are never explicitly asked to rate the personalities of the individuals. A control experiment rules out that these effects are merely due to an association of motor responses to individuals, and shows that a prior manipulation of vision–action fluency is critical to affect personal-trait judgments.

## 2 Experiment 1: Action movies

In Experiment 1, the participants were presented with movies of two individuals (‘George’ or ‘John’) carrying out either an academic action (typing on a keyboard) or a sporty action (kicking a ball). In a speeded response task, the participants had to identify the two persons by pressing either a finger or a foot key. Thus, the participant’s responses to identify a particular individual were either compatible with the sporty action carried out by this individual and incompatible with the academic action, or vice versa. We predicted that the compatibility between observed action and executed response should influence, first, the fluency with which the identification responses are executed; and second, the personality traits attributed to the two individuals.

### 2.1 Method

#### 2.1.1 Participants

Thirty-two students (27 females) ranging in age from 18 to 42 years participated in the study. All participants had normal or corrected-to-normal vision. The key assignment of actors (George/John) to response keys (foot/finger) was counterbalanced across participants. All participants filled out the Autism-Spectrum Quotient (Baron-Cohen, Wheelwright, Skinner, Martin, & Clubley, 2001) before taking part in the experiment (see Table 1, row 1, for the mean AQ and the distribution in the current sample).

#### 2.1.2 Material and apparatus

**Fig. 1:**
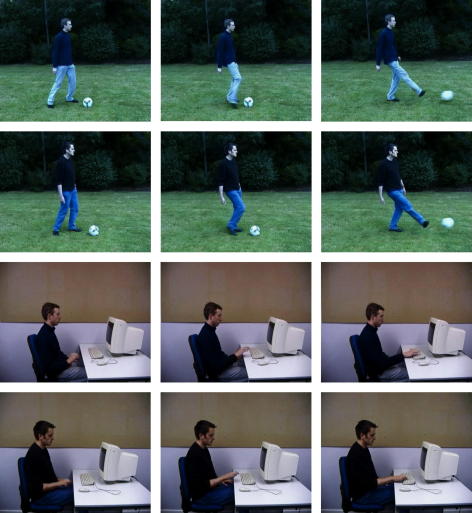
Examples for the movies used in Experiment 1 (action movies). The upper two rows show the ‘sporty’ kicking actions carried out by the two individuals. The lower two rows show the ‘academic’ typing actions carried out by the two individuals.

The experiment was controlled by Presentation run on a 3.0 GHz PC running Windows XP. Eight movies made up the stimulus set (see Fig. 1 for examples). The movies lasted 1100 ms each and subtended 5° visual angle vertically and 8° horizontally, given an average viewing distance of 60 cm. Two of these movies showed John or George kicking a football, and two movies showed John or George hitting a key on a computer keyboard. In these four movies, the movement direction was always from left to right. To exclude possible confounds arising from compatibility of movement direction and response, for each of these four movies a mirror-inverted version was created, in which the movement direction was from right to left.

#### 2.1.3 Procedure and design

The participants were seated in a dimly lit room facing a color monitor at a distance of 60 cm. After the computer-driven instruction and a short training phase of 16 trials the experiment properly started. It lasted for about 15 min and consisted of 320 trials. The eight different movies were presented at equal rates in a randomized order. Thus, there were 160 trials in which the actor had to be identified by a finger response. In these trials, he was equally often typing on a computer keyboard (compatible) or kicking a football (incompatible). In the remaining 160 trials, the actor had to be identified with a foot response. In these trials, he was again either kicking a ball (compatible) or typing on the computer keyboard (incompatible).

The course of each trial was as follows: After the participants initiated the trial by pressing the space bar with their left hand, the movie was presented after 500 ms. They identified John or George by either pressing the foot pedal with their right foot or the enter button on the computer keyboard with their right index finger. Participants were instructed to give their judgment in the interval in which the movie was on the screen (1100 ms). If their judgment was correct, the next trial started. If they committed an error or did not react in the given response interval of 1100 ms, an error message was displayed.

After the experiment was finished, a short questionnaire was presented on the computer screen. The participants had to indicate on a scale from −4 (‘not at all’) to 4 (‘very much’) how sporty they imagined the two actors to be (presented by name and an image of their face). They answered the same question with regard to whether they imagined the two individuals to be academic persons or not. The order in which these questions were presented was counterbalanced across participants (i.e., whether they rated George or John first and whether they gave ratings of sporty-ness before academic-ness).

### 2.2 Results

#### 2.2.1 Vision–action fluency

**Fig. 2:**
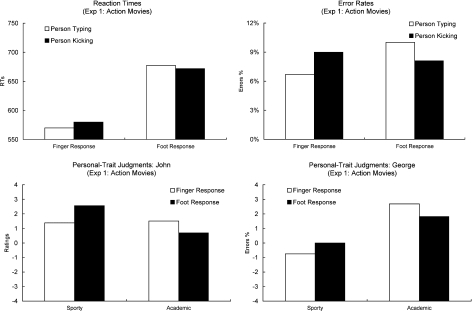
The upper two panels show the vision–action compatibility effects obtained in Experiment 1 (action movies) for RTs (upper left panel) and Error rates (upper right panel). The bars on the left show the data when an individual had to be identified with a finger response and the bars on the right show the data when an individual had to be identified with a foot response. The white bars show the data when the person was presented typing on a keyboard. The black bars show the data for when the person was kicking a football. The lower two panels show the person-trait judgment effects obtained for John (lower left panel) and George (lower right panel). The bars on the left show the ratings of how sporty a person appeared. The bars on the right show how academic a person appeared. The white bars show the data for when the person was identified by a finger response. The black bars show the data for when the person was identified by a foot response.

For the analysis of RTs (Fig. 2, upper left panel), trials in which the participants pressed the wrong button or did not react in the given reaction time interval were excluded (8%). The remaining RTs were entered into a repeated measurements ANOVA with the within-subjects factors Response (foot/finger) and Observed Action (kicking/typing). A main effect of Response was obtained (*F* [1, 31] = 175.2, *p* < .0001, *partial eta squared* = .85). Participants were faster in responding with the finger than with the foot. There was no main effect of Observed Action (*F* [1, 31] < 1). Individuals were identified equally quickly when they were presented typing or kicking. Finally, the predicted two-way interaction of Response and Observed Action was marginally significant (*F* [1, 31] = 3.0, *p* = .093, *partial eta squared* = .09). When identifying an individual with a foot response, RTs were faster when the person was seen kicking a ball than when typing. When identifying an individual with a finger key-press, RTs were faster when viewing a typing action than when viewing a kicking action.

The analysis of the Error Rates (Fig. 2, upper right panel) did not reveal main effects for Response (*F* [1, 30] < 1) or Observed Action (*F* [1, 31] < 1). Therefore, foot and hand responses were equally accurate and the individuals were identified equally reliably when they were presented typing or kicking. However, the interaction of Response and Observed Action was highly significant (*F* [1, 31] = 9.2, *p* = .005 *partial eta squared* = .23). Hand responses were more accurate when typing actions were observed than when kicking actions were observed. Conversely, foot responses were more accurate when kicking actions were observed than when typing actions were observed.

#### 2.2.2 Personal-trait judgments

Before analyzing the data of the personal-trait judgment task (Fig. 2, lower panels), we checked whether the two persons were rated differently on the two traits. To this end, the data were entered into a repeated measures ANOVA with the factors Person (John, George) and Trait (academic, sporty). There was a main effect of Trait (*F* [1, 31] = 16.0, *p* < .0001, *partial eta squared* = .34) and a main effect of Person (*F* [1, 31] = 7.0, *p* = .012, *partial eta squared* = .19). Overall, the two individuals were rated to be more academic than sporty, and John generally received higher ratings than George. However, there also was an interaction of Person and Trait (*F* [1, 31] = 22.2, *p* < .0001, *partial eta squared* = .42). Thus, the two persons were rated differently on the two traits. John was judged sportier than George, but less academic than George.

Note that these differences had to be eliminated in order to obtain a pure measure of the effect of Response on the personal-trait judgments. That is, for each participant, the difference between the person identified with a foot response and the person identified with a finger response also reflects differences that are intrinsic to the two individuals, which had to be identified with these responses. Therefore, from each participant’s rating of the two people on each trait, the mean rating of this person on this trait across all participants was subtracted. This procedure eliminated all differences between the two individuals on the two traits but preserved the effects of Response on the personal-trait judgments. The data were then entered into a two-way ANOVA with the within-subjects factors Trait (academic/sporty) and Response (person identified with a finger response/foot response). There was no main effect of Response (*F* < 1), showing that the two persons were rated equally irrespective of whether they were identified with a foot or a finger response. Due to the normalization procedure described above, the effect of Trait was eliminated (*F* = 0). However, the predicted interaction of Trait and Response was significant (*F* [1, 31] = 7.4, *p* = .011, *partial eta squared* = .19). Thus, a person was judged more academic when he was identified with a finger response compared to when he was identified with a foot response. A person appeared sportier when he was identified with a foot response compared to when he was identified with a finger response.

### 2.3 Discussion

The present experiment demonstrated for the first time that the compatibility between observed actions with to be produced responses affects not only the fluency of the participants’ responses, but also the attribution of personal traits to the observed individuals. Foot-key responses were more fluent when the identified person was carrying out the sporty action (kicking a ball) compared to when he was carrying out the academic action (typing). This person was later judged to be sportier. Finger-key responses were more fluent when the identified person was carrying out the academic action compared to when the person was carrying out the sporty action. This person was perceived to be more academic. These vision–action personal-trait effects were observed for the ratings of both John and George, although the two individuals were rated quite differently (John was rated more sporty than academic; George was rated more academic than sporty).

It is essential to confirm in further experiments that the present effects on personal-trait judgments were indeed caused by the prior manipulation of vision–action fluency. Participants have been exposed to equal numbers of sporty and academic scenes for each individual. Therefore, the results are not an artifact of stimulus exposure. However, each participant always pressed the same key to identify a particular person. Consequently, one person could have become associated with a foot response, while the other person was associated with a finger response over the course of the first part of the experiment. The bias to sporty or academic would then be due to an association of motor responses with individuals, if one assumes that foot responses are more strongly associated with the sporty trait than finger responses, and vice versa for the academic trait. According to this account, the association of individuals and motor responses is sufficient to bring about changes in person perception; a prior induction of vision–action fluency is not required. Experiment 2 addresses this alternative explanation.

## 3 Experiment 2: Static images without action

The participants had the same task as in Experiment 1 (identify one person with a finger-key response and the other with a foot-key response), but overt action was removed from the stimuli. The two persons (John or George) were presented as static images either standing next to a ball (but not kicking it), or sitting next to a computer keyboard (but not typing). Thus, the sporty and academic contexts of the scenes were comparable to Experiment 1, but because no overt action was presented, the vision–action fluency effects that arose from the compatibility between observed actions and to be executed responses should be eliminated.

This modification of the original paradigm allowed us to address the alternative explanation that the effects on personal-trait judgments were due to an association of motor responses with individuals. If this were the case, then the same effects as in Experiment 1 should be observed because the association of individuals to motor responses was also the same as in Experiment 1. If, however, the effects on personal-trait judgments were due to a prior induction of vision–action fluency, then a reduction of the vision–action fluency effects should lead to similar reductions of the effects on personal-trait judgments.

### 3.1 Method

#### 3.1.1 Participants

Thirty-two students (23 female) ranging in age from 20 to 30 years participated in the study. All participants filled out the Autism-Spectrum Quotient (Baron-Cohen et al., 2001) before taking part in the experiment (see Table 1, row 2, for the mean AQ and the distribution in the current sample). All other aspects of the participant selection were as in the previous experiment.

#### 3.1.2 Material and apparatus

**Fig. 3:**
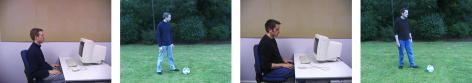
Stimuli used in Experiment 2 (static images without action). From left to right: George sitting next to a keyboard, George standing next to the football, John sitting next to a keyboard, and John standing next to a football.

The apparatus was identical to that of the previous experiment. The material comprised eight static images of the individuals (John or George) standing or sitting next to the objects instead of the action movies of the previous experiment (see Fig. 3 for examples). Again, the people could either be facing to the left or to the right. Visual angles and exposure times were identical to Experiment 1.

#### 3.1.3 Procedure and design

The experimental setup and the course of each trial were identical to the previous experiment.

### 3.2 Results

#### 3.2.1 Vision–action fluency

**Fig. 4:**
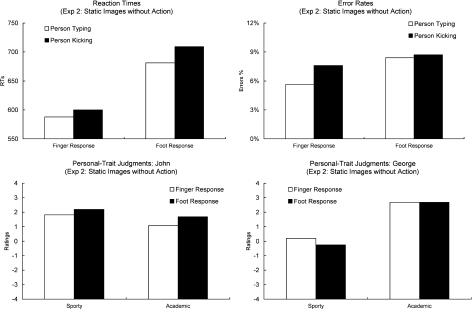
The upper two panels show the vision–action compatibility effects obtained in Experiment 2 (static images without action) for RTs (upper left panel) and Error rates (upper right panel). The bars on the left show the data when an individual had to be identified with a finger response and the bars on the right show the data when an individual had to be identified with a foot response. The white bars show the data when the person was presented typing on a keyboard. The black bars show the data for when the person was kicking a football. The lower two panels show the person-trait judgment effects obtained for John (lower left panel) and George (lower right panel). The bars on the left show the ratings of how sporty a person appeared. The bars on the right show how academic a person appeared. The white bars show the data for when the person was identified by a finger response. The black bars show the data for when the person was identified by a foot response.

The reaction time (Fig. 4, upper left panel) and error data (Fig. 4, upper right panel) were analyzed as in Experiment 1. For the analysis of the RTs, trials in which the participants pressed the wrong button or did not react in the given reaction time interval were excluded (7%). The ANOVA revealed a main effect of Response (*F* [1, 31] = 247, *p* < .0001, *partial eta squared* = .89). Participants were faster in responding with the finger than with the foot. There also was a main effect of Observed Action (*F* [1, 31] = 27.0, *p* < .0001, *partial eta squared* = .47). Participants identified the individuals more quickly when they were presented next to a keyboard than when they were presented next to a football. Importantly, in contrast to Experiment 1, there was no interaction of Observed Action and Response (*F* [1, 31] = 2.6, *p* = .12, *partial eta squared* = .08). However, because the *p*-value was close to significance it is important to note that this trend for an interaction is in the opposite direction to that found in Experiment 1. That is, responses were faster to identify the individuals seen sitting adjacent to a keyboard, and this advantage was *larger* when making a foot response. This is the opposite pattern to that expected, and observed in Experiment 1, based on vision–action fluency/priming.

The analysis of the Error Rates revealed a main effect of Response (*F* [1, 31] = 6.2, *p* < .05, *partial eta squared* = .17), showing that hand responses were more accurate than foot responses. There was no main effect for Observed Action (*F* [1, 31] = 2.0). As in RTs, the critical two-way interaction between Response and Observed Action was not significant (*F* [1, 31] < 1, *partial eta squared* = .02).

#### 3.2.2 Personal-trait judgments

The data for the personal-trait judgment task (Fig. 4, lower panels) were analyzed as in Experiment 1. The analysis of the differences between the ratings of John and George replicated the findings of Experiment 1. There was a main effect of Trait (*F* [1, 31] = 18.4, *p* < .0001, *partial eta squared* = .19) and a main effect of Person (*F* [1, 31] = 7.5, *p* = .01, *partial eta squared* = .37). Accordingly, the two individuals were rated to be more academic than sporty, and John generally received higher ratings that George. Again, there also was an interaction of Person and Trait (*F* [1, 30] = 40.9, *p* < .0001, *partial eta squared* = .57) reflecting that John was perceived sportier than George, but less academic.

These differences were again eliminated from the data to assess the effects of Response on the sporty and academic ratings. This analysis revealed no main effect of Response (*F* < 1) and Trait (*F* = 0). In contrast to Experiment 1, there also was no interaction of Trait and Response (*F* [1, 31] = 2.4, *ns*, *partial eta squared* = .04). If anything, the data showed the reverse pattern to Experiment 1. Persons identified with a foot response were judged slightly less sporty than persons identified with a finger response. Persons identified with a finger response were judged slightly less academic than persons identified with a foot response.

### 3.3 Discussion

There were no effects of vision–action fluency in either the RTs or the Error rates. Likewise, the personal-trait judgments were not affected by the motor response (foot, finger) that was required to identify the individuals. This was the case even though the participants were efficiently rating the individuals and reproduced the general bias of John being rated as sportier, and George as more academic. Therefore, the present experiment confirmed that the mere association of motor responses (foot or finger) to individuals when identifying them did not suffice to affect personal-trait judgments, but that a prior manipulation of vision–action fluency is critical.

The results of Experiment 2 also supported the idea that the vision–action fluency effects in RTs and error rates in Experiment 1 reflected the compatibility of perceived actions with the responses required to identify the individuals. The mirror system is preferentially activated for actions directed at objects if biological motion is present in the stimuli (Tai, Scherfler, Brooks, Sawamato, & Castiello, 2004). Consistently, when biological motion and all cues for action were eliminated, foot and finger responses were equally fluent irrespective of whether the individuals were presented in the academic or sporty contexts. The presence of objects and effectors that were also present in Experiment 1 did not affect the fluency of the participants’ responses.

Please note that the present study did not allow us to rule out that the absence of biological motion by itself was critical to eliminate the vision–action fluency effects. For instance, the motion cues in Experiment 1 could have drawn attention to critical body parts of the individuals (feet, hands) or to the critical objects in the scenes (football, computer keyboard). Thus, the vision–action fluency effects observed in Experiment 1 could also have reflected interactions between the participants’ responses and either the compatible objects (e.g., Tucker & Ellis, 1988, 2001) or compatible body parts (Reed & Farah, 1995). But note that both of these notions imply that the action system of the observers was affected by the stimuli they perceived. Thus, they do *not* challenge the view that the personal-trait judgment effects depended on the prior fluency states of the participants’ action system while identifying the individuals.

## 4 Experiment 3: Action movies, implicit personality assessment

In Experiment 1, the participants were required to make explicit decisions by rating the personal traits (sporty/academic) of the individuals they had observed earlier in the experiment on a scale. However, many social cognitive processes do not take place in such an explicit way, and many processes may not be available to conscious/explicit access. In addition, it is important to show that the attribution of the personal traits occurs not only when the participants are required to do so, but spontaneously while the participants observed the acting individuals. Therefore, in this experiment we assessed the personal-trait effects with an implicit measure, where the participants were never asked to make personal-trait judgments, and where they had no knowledge that such an issue was investigated.

The first part of the experiment was identical to Experiment 1, but afterwards the participants were *not* given the short questionnaire that explicitly required them to attribute the traits ‘sporty’ and ‘academic’ to the observed individuals. Instead, the personal-trait judgment effects were now assessed with a priming task. The participants were instructed to categorize a variety of scenes as to whether they were sporty or academic. The scenes were preceded by brief presentations of the faces of either John or George, which the participants were instructed to ignore. If the traits ‘sporty’ and ‘academic’ had become associated with the two individuals while the participants were identifying them, then the faces of the two individuals should now act as a prime and affect the identification of the scenes. More specifically, it should be easier to categorize a scene as ‘sporty’, when the face of the person that was identified with a foot response was presented beforehand. In this case the participants’ responses were more fluent whenever this person carried out the sporty action than when carrying out the academic action. Analogously, a scene should be more easily categorized as ‘academic’ when the face of the person that was identified with a finger response was presented beforehand.

In contrast, if the effects on personal-trait judgments in Experiment 1 only occurred because the participants were explicitly required to make such judgments, there should now be no effects of the face-primes on the categorization of the scenes.

### 4.1 Method

#### 4.1.1 Participants

Thirty-two students (26 female) ranging in age from 20 to 30 years participated in the study. All participants filled out the Autism-Spectrum Quotient (Baron-Cohen et al., 2001, see Table 1, row 3, for the mean AQ and the distribution in the current sample) before taking part in the experiment. All other aspects of the participant selection were as in the previous experiments.

#### 4.1.2 Material and apparatus

**Fig. 5:**
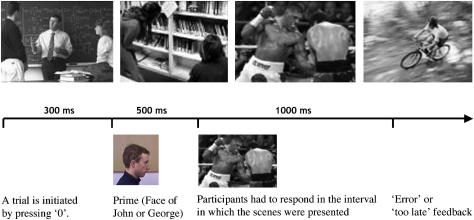
The upper four panels show examples of the stimuli used in the implicit person priming task (Experiment 3, action Movies, implicit personality assessment). From left to right: two academic scenes and two sporty scenes. The lower half shows the time course of the trials in the implicit personal-trait judgment task of Experiment 3.

The material and apparatus used in the first part of this experiment, where video clips of George and John were identified with finger or foot responses, were identical to experiment one. In the second part of the experiment, 24 new photographs were used. Four images were profile shots of the faces of John and George, facing either to the left or the right (visual angle: 2° horizontally, 2° vertically). The remaining 20 black-and-white photographs were shots of 10 sporty and 10 academic scenes (see Fig. 5 for examples). The horizontal and vertical visual angles of these images varied between 2° and 3°.

#### 4.1.3 Procedure and design

The first stage of the experiment, where individuals were identified with finger and foot responses, was identical to that of experiment one. After completion of this stage, the participants carried out another short experiment, lasting about 5 min and consisting of 80 trials. In this experiment, the faces of either John or George were presented for 500 ms at equal rates and in random order. Immediately afterwards, one of the academic or sporty scenes was presented for 1000 ms (see Fig. 5 for the time course of the trials). Participants were instructed that the face images were now irrelevant to their current task and so should be ignored. Rather, their task now was to rapidly classify the subsequent scene as sporty or academic by pressing the ‘1’-key or ‘7’-key on the keyboards numerical block with their left hand. If they pressed the wrong button or failed to react in the interval of 1000 ms, a short error message was displayed. Otherwise, the next trial started after the participants had pressed the zero-key on the numerical key-block with their right index finger.

### 4.2 Results

#### 4.2.1 Vision–action fluency

**Fig. 6:**
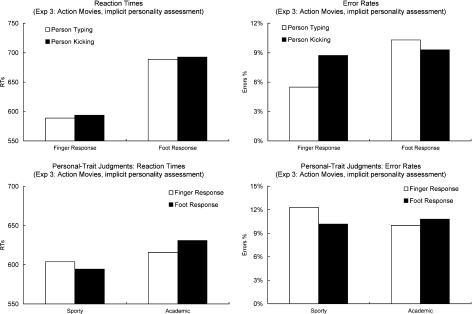
The two upper panels show the vision–action fluency effects obtained in Experiment 3 (action Movies, implicit personality assessment) for RTs (upper left panel) and Error rates (upper right panel). The bars on the left show the data when an individual had to be identified with a finger response and the bars on the right show the data when the individual had to be identified with a foot response. The white bars show the data when the individuals were presented typing. The black bars show the data when the individuals were presented kicking a ball. The lower two panels show the RTs (left panel) and Error rates (right panel) in the implicit personality-priming task (Experiment 3). The bars on the left show the data for the identification of sporty scenes and the bars on the right show the data for the identification of the academic scenes. The white bars show the data for scenes preceded by a photograph of the person that had to be identified with a finger response. The black bars show the data for scenes preceded by the image of the person that had to be identified with a foot response.

The analysis of the vision–action fluency effects in RTs (Fig. 6, upper left panel) and Error Rates (Fig. 6, upper right panel) was carried out as in the previous experiments. Trials in which the participants pressed the wrong button or did not react in the given reaction time interval were excluded from the analysis of the RTs (8%). As in Experiment 1, the analysis of RTs revealed no main effect of Observed Action (*F* [1, 31] = 1.1, *partial eta squared* = .03). The individuals were identified equally quickly when they were carrying out typing or kicking actions. There was a main effect of Response (*F* [1, 31] = 330.2, *p* < .0001 *partial eta squared* = .91). Participants were faster in responding with the hand than with the foot. The two-way interaction of Response and Observed Action was not significant (*F* [1, 31] < 1, *partial eta squared* = .01).

The analysis of the Error Rates showed a main effect of Response (*F* [1, 31] = 6.9, *p* = .013, *partial eta squared* = .22). Finger responses were more accurate than foot responses. There also was an effect of Observed Action (*F* [1, 31] = 5.9, *p* < .021, *partial eta squared* = .16). The individuals were identified more reliably when they were presented typing than when they were presented kicking a ball. Finally, the critical interaction between Response (finger/hand) and Observed Action (kick/type) was again significant (*F* [1, 31] = 5.3, *p* < .028, *partial eta squared* = .15). Hand responses were more accurate when typing actions were observed than when kicking actions were observed. Foot responses were performed more accurately when kicking actions were observed than when typing actions were observed.

#### 4.2.2 Personal-trait judgments

Fig. 6 shows the RTs (lower left panel) and errors rates (lower right panel) to categorize the pictures as sporty or academic in the priming procedure. Trials in which the participants pressed the wrong button or did not react in the given reaction time interval of 1000 ms were excluded (11%) from the analysis of RTs. The remaining data were entered into a repeated measures ANOVA with the within-subjects factors Response (person identified with a finger response/person identified with a foot response) and Scene (academic scene/sporty scene). It revealed a main effect of Scene (*F* [1, 31] = 16.9, *p* < .0001, *partial eta squared* = .36). In general, the participants were faster in classifying sporty scenes than academic scenes. There was no main effect of Response (*F* < 1), but an interaction of Response and Scene (*F* [1, 31] = 4.3, *p* = .046, *partial eta squared* = .12). The participants were faster in classifying a scene as academic when the person identified with a finger response was presented beforehand than when the person identified with a foot response was presented beforehand. Conversely, the participants were faster in classifying a sporty scene when the person identified with a foot response was presented beforehand than when the person identified with a finger response was presented beforehand.

The Error Rates (Fig. 6, lower right panel) were analyzed with the same ANOVA. However, no significant effects were obtained (for all, *F* < 1.9). It is noteworthy, however, that the Error Rates show exactly the same pattern as the RTs. The participants were more accurate in identifying an academic scene when the person identified with a finger response was presented beforehand than when the person identified with a foot response was presented beforehand. Conversely, the participants identified a sporty scene more reliably when the person identified with a foot response was presented beforehand than when the person identified with a finger response was presented beforehand.

We investigated the significant RT priming effects further by examining the effects in the first and second half of the procedure. It is possible that the person-trait priming effects are stronger in the initial trials because: (a) participants habituate to the irrelevant priming faces with repeated exposures; (b) the person-trait effect is transient, only lasting a few minutes after the vision–action matching processes; and (c) as RTs to categorize the scenes get substantially faster with repeated exposure, priming effects might get smaller. Therefore, we analyzed separately the first 40 trials and the second 40 trials of the implicit personal-trait priming task. This analysis showed that the interaction of Person and Scene was highly significant in the first half of the personal-trait priming task (*F* [1, 31] = 5.1, *p* < .032, *partial eta squared* = .14), but not for the second half (*F* < 1, *partial eta squared* = .01). The effect on error rates was neither significant in the first or the second half. However, numerically, the effect in the error rates was also stronger in the first than the second half.

### 4.3 Discussion

The vision–action fluency effects generally replicate those of Experiment 1. More importantly, the new priming measure for the vision–action personal-trait effect was also significant. Again, the personality of the observed individuals took on the properties of the action for which the participants’ responses were more fluent. Participants were quicker in judging a scene as ‘academic’ when they were primed with the face of the person that was more fluently identified when carrying out the academic action (typing) than when carrying out the sporty action (kicking a ball). Likewise, participants were quicker in judging a scene as ‘sporty’ when they were primed with the face of the person that was more fluently identified when carrying out the sporty action (kicking a ball). Consequently, the attribution of personal traits to individuals on the basis of vision–action fluency occurred even though the participants had no knowledge that this issue was investigated and the faces of John and George were irrelevant to the task of scene categorization. Moreover, the present findings support the view that the attribution of personal traits on the basis of vision–action fluency occurred spontaneously during the observation of the acting individuals, and not only when the participants were asked to do so at the end of the experiment.

It is worth noting that the personal-trait priming effects were much clearer in the first half of the priming procedure. As noted, there are a number of possible reasons for this result. First, it may be the case that with repeated exposures to the faces participants habituate to them, hence less encoding would produce no facilitation effects. Second, it may be the case that the personal-trait associated with an individual via previous vision–action fluency is transient. Hence after a few minutes the effect might dissipate and third, it could simply be the case that participants are able to very rapidly encode and categorize the sport/academic scenes after repeated exposure to them, and such ‘ceiling’ performance reduces the likelihood of detecting any priming effects. Further work will be necessary to decide between these alternatives.

## 5 Interindividual differences

The aim of this final section was twofold. The first aim was to investigate the hypothesis that autism could be characterized by a mirror neuron dysfunction, that is, a deficit in mapping perceived actions to one’s own action representations (e.g., Oberman, Hubbard, McCleery, Ramachandran, & Pineda, 2005; Williams et al., 2001). Importantly, however, other researchers have argued that autistic individuals were ‘far from action blind’ (Sebanz, Knoblich, Stumpf, & Prinz, 2005) and that brain structures interacting with the mirror system were sub-optimal in autism (e.g., Arbib & Yahya, 2002). According to this latter view, autistic individuals might be impaired only in the ‘use’ of information provided by intact vision–action matching systems.

To differentiate between these two possibilities, we analyzed the results from Experiments 1 to 3 with regard to the data from the Autism-Spectrum Quotient (Baron-Cohen et al., 2001) filled out by all participants prior to taking part in the experiments. The distribution of the current sample corresponded well to the non-clinical control group assessed by Baron-Cohen and colleagues (see Table 1, for the distribution of the scores in the current samples).

The AQ presupposes that autism was an extreme case of typical variations in social-communication disability, and represents healthy and more autistic individuals on a continuous scale, with higher scores for individuals closer to the autism end of the scale. The AQ is derived from 50 different questions that reflect the five ‘traits’ associated with autism (10 questions each: social skill, attention switching, attention to detail, communication, and imagination). The AQ has been shown to be a valid and reliable measure for autistic ‘traits’ and corresponds well to clinical diagnoses of autism (Austin, 2004; Baron-Cohen et al., 2001). It also traces known autistic deficits of social cognition such as eye-gaze cuing in the behavior of normals (Bayliss & Tipper, 2005; Bayliss, DiPellegrino, & Tipper, 2005).

Thus, if autism is associated with damage to the mirror system, then persons with more autistic traits should (1) show smaller effects of vision–action fluency in the first part of the experiment and (2) show reduced effects on personal-trait judgments. If, however, more autistic individuals were impaired only in the use of the information provided by the vision–action matching processes, then a participant’s AQ-score should not influence the induction of vision–action fluency in the first part of the experiment, but should influence whether he shows effects in the personal-trait judgments.

The second aim was to confirm our interpretation that the effects on personal-trait judgments were due to a prior induction of vision–action fluency. If this were the case, then there should be a positive relationship between the induction of vision–action fluency in the first parts of the experiments and subsequent effects on personal-trait judgments. Those participants that were the most affected by the compatibility of perceived actions and to be produced responses should also exhibit the strongest effects on personal-trait judgments.

To investigate both questions, we carried out regression analyses for each of the experiments. For each experiment, the participants’ AQ-scores were entered as predictors in separate regression analyses (stepwise method), (1) for the induction of vision–action fluency in the first part of the experiments, and (2) the effects on personal-trait judgments in the second parts. The participants’ effect on vision–action fluency was entered as a second predictor in the analysis of the personal-trait judgment effects.

### 5.1 Method and results

#### 5.1.1 Vision–action fluency

For each experiment, the average vision–action fluency effect in the first part of the experiment was calculated for each participant, separately for the Error Rates and RTs (i.e., the difference between RTs/Error Rates when responses and irrelevant visual-actions were compatible and when responses and irrelevant visual-actions were incompatible). These values were then entered as dependents into two separate linear regression analyses (stepwise method) for the RTs and Error Rates with the participants’ AQ-scores as single predictors. Table 2 shows the result of this analysis. The participants’ AQ-scores were not a significant predictor for the participants’ vision–action fluency effect in any of the experiments for the RTs or the Error Rates. Thus, autism and vision–action matching (mediated by mirror systems) do not seem to be related in this study.

#### 5.1.2 Personal-trait judgments

For the analysis of the effects on personal-trait judgments, the predictors were the participants’ AQ-score and the vision–action fluency effect in RTs and Error rates. The dependent variable that described a participant’s effect on personal-trait judgments was computed in the following way: for Experiments 1 and 2, the average of the sporty judgments for the person identified with the foot-key was subtracted from the academic ratings for this person, and the academic judgments for the person identified with the finger-key were subtracted from the sporty ratings for this person. For Experiment 3, the effect in RTs in the first 40 trials, for which the personal-trait judgment effect was significant, was used as a dependent variable. Here, it was derived by subtracting the responses to the scenes that were preceded by a ‘compatible’ face (academic scenes, person identified with a finger-key; sporty scenes, person identified with a foot-key) from those preceded by an ‘incompatible’ face (academic scenes, person identified with a foot-key; sporty scenes, person identified with a finger-key). Table 3 shows the results of these analyses.

This analysis revealed significant models for the personal-trait judgment effects in Experiment 1 (action movies: *R* = .59; *p* < .005) and Experiment 3 (implicit personal-trait judgments: *R* = .352; *p* < .05), but not in Experiment 2 (no action cues). In both Experiments 1 and 3, the vision–action fluency effects were a significant predictor of the subsequent effect on personal-trait judgments (Experiment 1: *r* = .404; Experiment 3: *r* = .352; both *p* < .05). Thus, the more a participant’s responses (foot/finger key to identify George/John) were affected by the irrelevant actions (kicking/typing), the more his/her personal-trait judgments were affected, irrespective of whether these judgments were measured explicitly or implicitly.

In addition, the AQ-score of the participants was a highly significant predictor of the effect on explicit personal-trait judgments in Experiment 1 (*r* = .533, *p* < .005). Surprisingly, this relationship was positive. Thus, the personal-trait judgments of participants with features more symptomatic of autism were even more affected by induction of vision–action fluency in the first part of the experiment. A similar relationship was, however, *not* observed in Experiment 3, in which the personal-trait judgment effects were measured implicitly.

### 5.2 Discussion

The regression analysis confirmed that the effects on personal-trait judgments were induced by the prior manipulation of vision–action fluency. That is, there was a significant positive relationship between the vision–action fluency effects in the first part of the experiment and the subsequent changes in personal-trait judgments in Experiment 1 and 3, but not in Experiment 2. Thus, the more the irrelevant actions influenced the fluency of a participant’s responses, the more personal-trait judgments were subsequently affected. The lack of such a relationship in Experiment 2 also supports the view that the effects on personality judgments were due to prior interactions of perceived actions and to be produced responses, and not due to interactions of perceived objects or effectors and to be produced responses. Even those participants whose responses were affected by either the perceived effectors, objects, or implied future actions in Experiment 2 did not show larger personal-trait judgment effects. Thus, any effect on personal-trait judgment seems to be driven by prior interactions of perceived actions and produced responses.

The results of the regression analyses contrasted, however, with the notion that autism was characterized by a mirror neuron dysfunction. If this had been the case, there should have been negative relationships between AQ-score and the induced vision–action fluency on the one hand, and between AQ-score and the effect on personal-trait judgments on the other hand. However, there was no relationship between AQ- score and vision–action fluency, and the relationship between AQ-score and effect on personal-trait judgments was positive.

These findings are consistent with the notion that the vision–action matching systems are not absent in autistic individuals, but that these individuals differ from more socially adept individuals in the use of the information provided by these systems. The finding of a positive relationship between the presence of autistic symptoms and the effects on personal-trait judgments suggests that persons with the highest AQ-scores in our (non-clinical) sample had more problems than the AQ-low-scorers with representing the actions of others independently from the actions they carried out at the same time.

Of potential importance, the personality-priming task in Experiment 3 appeared to be less sensitive to interindividual differences than the explicit measure in Experiment 1. Thus, when the personal-trait judgments were assessed implicitly in the priming procedure of Experiment 3, there was no relationship with the AQ-scores of the participants.

## 6 General discussion

The mirroring of the behavior of others is fundamental to fluent social intercourse. This capacity seems to rely on the matching of perceived actions to the corresponding action representations of the observer (e.g., Hommel et al., 2001; Rizzolatti et al., 2000). The subtle mimicking behavior that results from these processes might form the basis of observational learning, facilitates social interactions, and generates bonding and rapport between persons (e.g., Chartrand & Bargh, 1999).

The present work goes beyond these findings and provided evidence that action mirroring also plays a role in higher social cognitive functions. We have demonstrated that the compatibility between observed actions and to be executed responses can influence the attribution of personal traits to other individuals. In a task in which the participants had to identify two individuals by pressing either a finger-key or a foot-key, we manipulated whether these responses were compatible either with a sporty action (kicking a ball) carried out by an individual, or with an academic action (typing on a keyboard). We found that an individual was identified more fluently when the irrelevant action he was carrying out was compatible with the response required to identify him. Although the compatibility effects were generally more pronounced in the error rates than in the RTs, this result replicated prior reports of perceived actions facilitating compatible responses or interfering with incompatible responses (e.g., Brass et al., 2000; Kilner et al., 2003). Such effects are expected if perceived actions were automatically mapped onto the action representations an observer would rely on to carry out these actions (e.g., Hommel et al., 2001; Rizzolatti et al., 2000). Consistent with this view, there were no vision–action compatibility effects when the to be identified individuals were not carrying out an action (Experiment 2).

Importantly, the compatibility between observed actions and responses also affected the subsequent attribution of personal traits to the individuals. The personality of the two individuals took on the properties of the action for which they were identified more fluently. Two findings confirmed that the effects on personal-trait judgments were due to prior interactions of perceived actions and executed responses in the action system of the observer. First, the regression analysis of Experiment 1 revealed a direct relationship between the effects on personal-trait judgments and the vision–action fluency effects in the first part of the experiment. This relationship was replicated in Experiment 3, in which the personal-trait effects were assessed implicitly via a priming task. Therefore, the more a participant’s identification responses were affected by the irrelevant actions, the stronger was the influence on personal-trait judgments at a later point in time, irrespective of whether these personal-trait judgments were measured explicitly or implicitly.

Second, the personal-trait judgments were only affected when a prior induction of visuomotor fluency was successful (Experiments 1 and 3). When the observed individuals were not acting (Experiment 2), there was no evidence for vision–action compatibility, and of course, there were no personal-trait judgment effects, either. Note that even the regression analysis failed to reveal a significant relationship between induced vision–action fluency and subsequent personal-trait judgments in Experiment 2. Thus, even those participants who showed compatibility effects (for instance, because they attended to either the implied but not performed actions, the objects, or the effectors present in the scenes) did not show larger personal-trait effects than the participants who did not exhibit compatibility effects. Although preliminary, this finding suggests that the effect on personal-trait judgments depends on actions that are truly perceived, instead of other stimulus aspects that could, in principle, have evoked compatibility effects.

These findings support embodied or simulation accounts of social perception (e.g., Barsalou et al., 2003; Gallese, 2001, 2003; Gallese & Metzinger, 2003; Iacoboni, 2005; Preston & de Waal, 2002). Accordingly, observers recreate the bodily states of others on the basis of their own action system to gain information about the goals, emotional states, and personal traits of these persons. Thus, by simulating the observed actions of others, such as frowning or smiling, or vigorous or slow ponderous movements, the emotional state of another person may be better understood. Similarly, our personal-trait effects emerged from a process of vision–action matching, that is, an interaction between observing an incidental action that is irrelevant to the task at hand with the response to be produced when identifying an individual. For example, when George can be identified with a foot response more fluently when he is seen kicking a ball, he is associated with this sporty property.

Further studies are required to pinpoint the exact nature of the mechanism that evoked the personal-trait judgment effect. We predicted such effects if observers tended to misattribute the fluency of their own responses to the actions they have perceived at the same time. If this were the case, other manipulations that only affect the action system of the observers while identifying the individuals (e.g., making responses easier or harder in some situations) should lead to similar results on personal-trait judgments. However, there are other possibilities to explain the vision–action personal-trait effect. According to Hommel and colleagues (2001) there is not only an influence from perception on action, but also a reverse influence from action on perception. Thus, the performance of a foot press might have interfered with the perception of the incompatible typing action, and/or facilitated the perception of the compatible kicking action. Theorists of embodied (social) cognition offered a similar explanation for enhancing effects of mimicry on perceptual measures. Accordingly, mimicry provides additional activation to the representation of a compatible stimulus (e.g., Barsalou et al., 2003). These notions suggest a more perceptual origin of the effects. If this were the case, manipulations that affect only the fluency of the perceptual processes while identifying the individuals (e.g., increasing contrast) should lead to similar effects on personal-trait judgments. Indeed, it has been demonstrated that manipulations that facilitated perceptual processing evoked more favorable judgments about the perceived stimuli (e.g., Mandler, Nakamura, & Van Zandt, 1987; Reber, Winkielman, & Schwartz, 1998; for a review of related findings, see Barsalou, 1999, 2003).

The present study also provided some preliminary information concerning the interindividual differences mediating the vision–action personal-trait effects. Participants completed the Autism-Spectrum Quotient developed by Baron-Cohen and colleagues (2001). This measures traits associated with autism, with higher scorers being closer to the autism end of the scale. The AQ has been shown to be a valid and reliable measure for autistic symptoms and corresponds well with clinical diagnoses of autism (Austin, 2004; Baron-Cohen et al., 2001). It should be noted, of course, that because only one of our participants had an AQ-score that fell in the range associated with a clinical diagnosis of autism, any conclusions we draw here must subsequently be confirmed with clinical studies.

We investigated two issues: first, whether the AQ-scores are related to the vision–action compatibility effects assumed to reflect mirror processing; and second, the new issue of whether the personal-trait effects differ in people with different AQ scores. In the former case, there is some debate as to whether individuals with autism have intact vision–action matching (mirror) systems. On the one hand, Williams and colleagues (2001) suggested that individuals with autism are impaired in representing the actions of others via mirror systems. On the other hand, others (e.g., Sebanz et al., 2005; Théoret et al., 2005) suggest that in some circumstances individuals with autism represent even task irrelevant actions of other people within their action system. Our results support this latter view. In both Experiments 1 and 3, where significant vision–action compatibility effects were observed, these effects were not related to the AQ-score of the participants.

In contrast, when examining whether AQ score affected the personal-trait effects, individual differences were detected. The higher a participant’s AQ-score, the stronger were the effects on personal-trait judgments he exhibited. Importantly, this relationship between AQ and personal-trait assessment was only observed in Experiment 1 where explicit/conscious measures were taken. When we measured the personal-trait associations implicitly via a priming technique, such that participants were unaware such information was being assessed (Experiment 3), no AQ differences were detected.

Our current working hypothesis is as follows: the initial processes that match perceived actions to the observer’s action system are intact also in the individuals with the highest AQ-scores in our (non-clinical) sample. Thus, the relatively rapid and automatic computations undertaken by mirror systems provide equivalent inputs to later systems. However, it is the ability to utilize this information that might differ in individuals with more autistic traits. In Experiment 1, persons closer to the autistic end of the AQ-scale were more affected by the vision–action fluency effects when they subsequently made conscious decisions about the personal traits of another person. Low AQ scorers, on the other hand, appeared to be able to more effectively discount/inhibit prior vision–action processes when making overt decisions about an individual’s personal traits. As noted, this ability of low AQ participants to discount prior processing is only observed when consciously manipulating information in the explicit personal-trait task of Experiment 1: all individuals responded similarly when implicit/pre-conscious processes were assessed in Experiment 3.

Other research would appear to be compatible with this line of thought. For example, individuals with autism tend to mimic the actions (echopraxia) and words (echolalia) of others irrespective of their own goals. Thus, the basic perception–action matching processes that result in the ability to mimic are not absent in autistic individuals, but the subsequent appropriate use of this information is lacking. Furthermore, they also confuse the personal pronouns of “I” and “You” (e.g., Kanner, 1943, 1946; for review see Tager-Flusberg, 2000). It follows that autistic individuals have problems with coordinating separate representations of self and other (Rogers & Pennington, 1991; Russell & Jarrold, 1999; Théoret et al., 2005; for a review, see Williams et al., 2001). Consistently, in false belief tasks, autistic children are more prone than healthy subjects to attribute their own knowledge about a situation to individuals that would not have this information (e.g., Baron-Cohen, Leslie, & Frith, 1985; Peterson & Bowler, 2000; for a review, see Frith, 2001). Even more relevant to our current findings, Russell and Jarrold (1999) demonstrated that in contrast to normally developing children and those with mild learning difficulties, children with autism had significant problems in differentiating their own from another person’s actions. Normal children are clearly aware of whether an action was produced by themselves, or whether they observed someone else produce a similar action. For individuals with autism their own actions and the actions of others they observe are not differentiated.

Our findings were consistent with this view. Accordingly, the explicit personal-trait ratings of those with the highest AQ-scores in our sample were most affected by the induction of vision–action fluency, because they had problems with keeping the representations of the actions carried out by the observed individuals separate from the representations of their own actions. That is, their own visual–action fluency when identifying an individual with a foot response while they are observed undertaking a sporty action, for example, is inappropriately assigned to the viewed person. Thus, the observed person is perceived to be a more fluent athlete. Of course, when measured implicitly it is not possible to separate self-action fluency from other person properties, as these are outside strategic control.

## 7 Conclusions

The present work links lower-level processes typically studied within the domains of visual psychophysics and motor control to higher-level cognitive processes in social cognition. Prior work has shown that priming participants with words associated with the elderly (e.g., ‘wrinkle’) can influence them to subsequently walk more slowly (Bargh, Chen, & Burrows, 1996). Our work has now shown the opposite effects of vision–action fluency influencing the attribution of personal traits.

Perhaps the most striking aspect of these processes was their automaticity. This was reflected in two ways: first, in all experiments, the actions (kicking/typing) carried out by the two individuals were completely irrelevant to the participant’s task of person identification. Second, the personality-trait judgment effects could be observed with both explicit measures where participants were asked to rate the sporty/academic nature of George and John on a scale, and when implicit measures were taken in a priming task where participants were never explicitly asked to rate the personalities of the individuals. Thus, the attribution of personal traits to individuals occurs spontaneously during the perception and production of actions, and the trait information associated with a person can be accessed rapidly and automatically even when the person’s identity and personality are not relevant to the task.

## Figures and Tables

**Table 1:** The AQ-scores of the participants in the three experiments

	Range	Mean/SD	Low	Intermediate	High
Experiment 1	8–27	16.3/4.8	23	9	—
Experiment 2	9–28	17.4/5.5	21	11	—
Experiment 3	7–36	15.8/6.2	24	7	1
					
Baron-Cohen	5–37	16.4/6.5	119	51	4

The second column shows the range of the scores in each experiment, and the third column shows the mean AQ-scores and standard deviations. The third, fourth, and fifth columns show how many of the participants in each experiment fell into the ranges in which few autistic traits were present (low), the range in which some autistic traits are present (intermediate, AQ-scores of 20+), and in the range that indicates autistic traits in a similar extent as in individuals with clinical diagnoses of autism (high, AQ-scores of 32+). Ranges were defined according to Baron-Cohen and colleagues (2001). The last row shows the corresponding data for the non-clinical control group tested in the original publication (Baron-Cohen et al., 2001).

**Table 2:** The bivariate correlations of the vision–action fluency effects in RTs (middle column) and Errors (right column) with the participants’ AQ-score (^∗^*p* < .10; ^∗∗^*p* < .05; ^∗∗∗^*p* < .005)

	RTs	Errors
Experiment 1	−.186	−.185
Experiment 2	−.121	−.003
Experiment 3	−.202	.154

**Table 3:** Result of the regression analysis

	Predictors	Correlations	Coefficients
Experiment 1	Fluency RTs	−.046	
	Fluency errors	.243^∗^	.404^∗∗^
	AQ-score	.483^∗∗∗^	.533^∗∗∗^
			
Experiment 2	Fluency RTs	−.087	
	Fluency errors	−.207	
	AQ-score	−.031	
			
Experiment 3	Fluency RTs	.261	
	Fluency errors	.352^∗∗^	.352^∗∗^
	AQ-score	.228	

The third row shows the bivariate correlations of the personal-trait judgment effects with the respective predictor variables. The right row shows the standardized beta values of the coefficients that were significant predictors of the personality effect (^∗^*p* < .10; ^∗∗^*p* < .05; ^∗∗∗^*p* < .005).
